# MicroRNA‐130b targets PTEN to induce resistance to cisplatin in lung cancer cells by activating Wnt/β‐catenin pathway

**DOI:** 10.1002/cbf.3331

**Published:** 2018-04-13

**Authors:** Qiang Zhang, Bin Zhang, Leina Sun, Qingna Yan, Yu Zhang, Zhenfa Zhang, Yanjun Su, Changli Wang

**Affiliations:** ^1^ Department of Lung Cancer Tianjin Medical University Cancer Institute and Hospital, National Clinical Research Center for Cancer Tianjin China; ^2^ Key Laboratory of Cancer Prevention and Therapy Tianjin China; ^3^ Tianjin Lung Cancer Center Tianjin China; ^4^ Department of Pathology Tianjin Medical University Cancer Institute and Hospital Tianjin China; ^5^ Tianjin's Clinical Research Center for Cancer Tianjin China

**Keywords:** cisplatin‐resistance, lung cancer, miR‐130b, PTEN, Wnt/β‐catenin

## Abstract

More and more studies indicate the relevance of miRNAs in inducing certain drug resistance. Our study aimed to investigate whether microRNA‐130b‐3p (miR‐130b) mediates the chemoresistance as well as proliferation of lung cancer (LC) cells. MTS assay and apoptosis analysis were conducted to determine cell proliferation and apoptosis, respectively. Binding sites were identified using a luciferase reporter system, whereas mRNA and protein expression of target genes was determined by RT‐PCR and immunoblot, respectively. Mouse xenograft model was used to evaluate the role of miR‐130b in cisplatin resistance *in vivo*. The rising level of miR‐130b in cisplatin resistance LC cell lines (A549/CR and H446/CR**)** versus its parental cell lines, indicated its crucial relevance for LC biology. We identified PTEN as miR‐130b's major target and inversely correlated with miR‐130b expression in LC. Moreover, excessive miR‐130b expression promoted drug resistance and proliferation, decreased apoptosis of A549 cells. Suppression of miR‐130b enhanced drug cytotoxicity and reduced proliferation of A549/CR cells both internally and externally. Particularly, miR‐130b mediated Wnt/β‐catenin signalling pathway activities, chemoresistance and proliferation in LC cell, which was partially blocked following knockdown of PTEN. These findings suggest that miR‐130b targets PTEN to mediate chemoresistance, proliferation, and apoptosis via Wnt/β‐catenin pathway. The rising level of miR‐130b in cisplatin resistance LC cell lines (A549/CR and H446/CR) versus its parental cell lines, indicated its crucial relevance for LC biology. Moreover, excessive miR‐130b expression promoted drug resistance and proliferation, decreased apoptosis of A549 cells. These findings suggest that miR‐130b targets PTEN to mediate chemoresistance, proliferation, and apoptosis via Wnt/β‐catenin pathway.

## 1 INTRODUCTION

As a serious malignant tumour disease, lung cancer is usually accompanied with strong clinical manifestation.^1^, ^2^ The average survival time of lung cancer patients lasts only for several months, even with specialized treatment combination of surgery, chemotherapy, and radiation therapy.^3^ One of the important causes for this extremely high mortality is the drug resistance in chemotherapy procedure.^4^ Therefore, in order to gain better results of lung cancer therapy, it is crucial to find effective ways to counter the drug resistance through exploring the underlying mechanisms of chemoresistance.^5^ A number of studies explored cisplatin, an efficient spectrum drug against cancer that is frequently applied in the treatment of various cancers in the place of lung, breast, bladder and brain, etc.^6^, ^7^ Cisplatin triggers cancer cell death by cross‐linking with the DNAs to suppress replication and transcription.^8^ However, extended records of administrating cisplatin caused great drug fastness in those cisplatin‐applied tumour cells.^9^, ^10^ In order to keep the effectiveness of the cisplatin treatment, it is imperative for lung cancer cells to maintain a steady level of sensitivity against it. Considering recent studies that demonstrated the correlation between cancer cells and resistance to cisplatin, we examined how miR‐130b affects the cisplatin‐resistance in lung tumour cells in our research.

MicroRNAs (miRNAs) are non‐coding RNA molecules with around 20 to 25 nucleotides that can lead to a downregulation of target proteins through the degradation of this mRNA or through translational inhibition, which play an important role in various malignancies.^11^, ^12^ Abnormal miRNA expression has been observed in both physiological and pathological processes multiple human cancers like proliferation, invasion, apoptosis, and chemotherapy resistance.^12^ MicroRNA‐130b‐3p (miR‐130b) targets CYLD to suppress growth of cells and induce programmed death in human gastric cancer cells.^13^, ^14^ Moreover, miR‐130b was recorded to be lifted in triple negative breast cancer tissue in comparison with adjacent healthy ones, and miR‐130b mediated CCNG2 that could be closely connected with the deteriorating development of the cancer in question.^15^ However, the role of miR‐130b in chemoresistance lung cancer cells is still unknown.

In this study, we aimed to explore the role of miR‐130b in cisplatin‐resistance lung cancer cells. The upregulation of miR‐130b was identified in cisplatin‐resistance lung cancer cells. We found that miR‐130b responds to cisplatin resistance through altering the targeted PTEN level and subsequence Wnt/β‐catenin pathway. The discovery of miR‐130b/PTEN being a new regulator that controls cisplatin‐resistance in lung cancer offers a fresh molecular insight that might be utilized in new therapy development for cisplatin resistance in lung cancer.

## 2 MATERIALS AND METHODOLOGY

### 2.1 Cultured cells and chemical reagents

Our study adopts the cell lines A549 and H446 from the American Type Culture Collection in Manassas. The cisplatin‐resistant A549/CR and H446/CR cells were derived by incubation with stepwise increasing cisplatin concentrations. The cells were routinely cultured in RPMI‐1640 medium plus 10% fetal bovine serum (Gibco, NY) in humidified 5% CO_2_ incubator with temperature of 37°C. Cisplatin was obtained from selleckchem. MiR‐130b inhibitor, miR‐130b mimic (miR‐130bm), or the appropriate negative controls (NC) of miRNA inhibitor (miR‐iNC) and miRNA mimic (miR‐NC) were brought from GenePharma (Shanghai, China).

### 2.2 Cell viability

Cells for experiments were cultured overnight in plates with 96 wells (4 × 10^3^ cells/well). Subsequently, MTS assay was carried out with Promega MTS assay kit operated in accordance with manufacturer's instructions. The Wallac Victor 1420 Multilabel Counter from Perkin‐Elmer was used to determine luminescence measure. Each assessment was done in triplicate with 3‐time repetition to ensure minimum deviation.

## 3 SIRNA AND TRANSFECTION

PTEN siRNA and control siRNA were obtained from Santa Cruz. A549 and H446 cells had been seeded in plates with 12 wells for exactly 1 day to reach 20% to 30% confluence and then gone through transfection with Lipofectamine 2000according to manufacturer Invitrogen's manual. PTEN plasmid was obtained from Addgene. The above cells in plates with 30% to 40% confluence after 24‐hour cultivation were transfected with PTEN or NC plasmid using Lipofectamine 2000 abiding by the producer (Invitrogen)'s instructions. To determine the influence of miR‐130b on chemosensitivity and programmed death of cells, lung cancer cells went through transfection with MiR‐130b inhibiter or miR‐130b mimic.

### 3.1 RNA extraction and qRT‐PCR

After transfection for 24 hours, the cells' total RNA was obtained with Trizol (Invitrogen, Carlsbad, CA) following producers' manual. The synthesis of cDNA was carried out with the RevertAid™ First Strand cDNA Synthesis Kit of Thermo Fisher Scientific Inc. The qRT‐PCR was carried out with Takara's SYBR Premix Ex Taq II kit in the Bio‐Rad's CFX96 real‐time PCR system. The RNA levels of target genes were standardized with that of β‐actin gene by the 2^−△△Ct^ way. Each reaction was conducted in triplicate and with 3‐time repetition to ensure minimum deviation.

### 3.2 Western‐blotting assay

Western blotting was conducted with antibodies for PTEN, β‐catenin, β‐catenin (Ser552), β‐catenin (Ser675) (Cell Signalling Technology, Beverly), and β‐actin (Santa Cruz Biotechnology, Santa Cruz).

### 3.3 Apoptosis analysis

Hoechst 33258 nucleic acid stain purchased from Invitrogen was used for nuclear staining for the analysis of apoptosis.^16^, ^17^ Annexin V/propidium iodide (PI) staining was conducted with annexin‐Alexa 488 (Invitrogen) and PI. Caspase activity was evaluated via the SensoLyte Homogeneous AMC Caspase‐3/7 Assay Kit (Anaspec).

### 3.4 Luciferase reporter analysis

To determine the impact of miR‐130b, the 3′ UTR of PTEN was magnified and implanted into the downstream of the luciferase reporter gene in the pMIR‐REPORT luciferase reporter vector. The mutant 3′UTR of PTEN was magnified by applying wild PTEN 3′UTR as the template, and the mutant plasmid was obtained through Site‐Directed Mutagenesis Kit. Cells went through co‐transfection with miR‐130bm and wild PTEN 3′UTR, together with Renilla luciferase pRL‐TK vector as a control. After being transfected for 24 hours, cells were lysed with RIPA buffer. Luciferase activity was evaluated consequently via Dual Luciferase Assay System (Promega) following producer's manual.

### 3.5 Xenografts

The experiments related to animals were conducted under the Guide for the Care and Use of Laboratory Animals and were carried out following the institutional ethical principles for animal experiments. The research obtained the approval from the Committee on the Ethics of Animal Experiments of Tianjin Medical University Cancer Institute and Hospital. To process the stable A549 cells that overexpressed miR‐130b, we generated the recombinant lentivirus which has miR‐130b precursor sequence and empty viral vector (EV) in A549 cells. Routine A549 cells were transfected with 5 × 10^5^ units of lentivirus and then 1 μg/mL puromycin selectively for 2 weeks. The transfected A549 cells were harvested for animal experiments later. The cells were put in 100‐μL PBS with the concentration of 4 × 10^6^ cells/mL and administrated into the side of the BALB/C female athymic mouse that aged 5 to 6 weeks (*n* = 6). The administration of cisplatin was done intraperitoneally twice a week (3 mg/kg). A549 and A549/CR cells were put in 100‐μL PBS with the concentration of 4 × 10^6^ cells/mL and administrated into the side of BALB/C female athymic mouse aged 5 to 6 weeks (*n* = 6). Cisplatin (3 mg/kg), polyplex containing 500 nM miR‐21 inhibitor in 50 μL of PBS, or their combination was applied via intraperitoneal injection twice a week. The development of tumour was closely monitored by callipers, and its volumes were calculated with the formula of 1/2 × length × width.^2^ Mice were euthanized once tumours have grown into ~1.0 cm^3^ or larger. After dissection, tumour sections were put in 10% formalin and embedded in paraffin, which then went through TUNEL immunostaining with an AlexaFluor 488‐conjugated secondary antibody (Invitrogen) to detect the signals.

### 3.6 Statistical analysis

The analysis was conducted with GraphPad Prism V (Graphpad Software, CA, USA), and all figures were recorded as means ± SD. A *t*‐test was used to compare among various groups. *P* < 0.05 was viewed as statistically significant.

## 4 OUTCOME

### 4.1 Cisplatin‐resistant A549 and H446 cell generation

**Figure 1:**
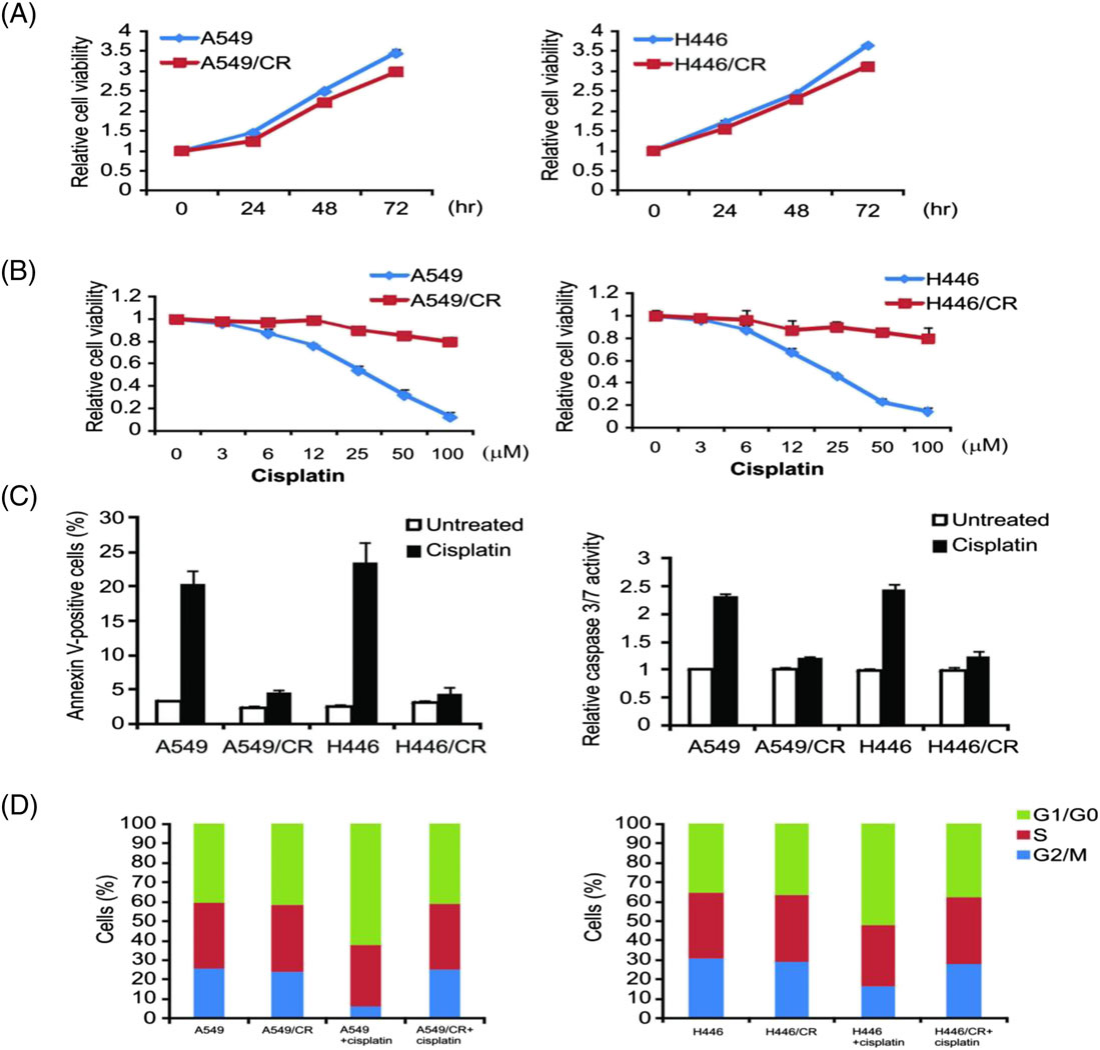
Generation of cisplatin‐resistant A549 and H446 cells. (A) Cell proliferation curves of A549/CR and H446/CR and the parental cells determined by MTS assay. (B) The indicated cell lines were treated with increasing concentrations of cisplatin for 72 hours. Cell proliferation was determined by MTS assay. (C) The indicated cell lines were treated with 40‐μM cisplatin for 24 hours. Apoptosis was analysed by Annexin V/PI staining followed by flow cytometry (left). Caspase 3/7 activity was determined by fluorogenic analysis (right). (D) The indicated cell lines were treated with 20‐μM cisplatin for 24 hours. Cell cycle distribution was detected by flow cytometry. Results in (A), (B), and (C) were expressed as means ± SD of 3 independent experiments

For exploring the mechanism of cisplatin‐resistance in lung cancer, we exposed A549 and H446 cells to cisplatin with rising levels of concentration for over 1 year. The cells that survived from each session with >70% cell confluency were passed through trypsinization, which leads to an elevated level of cisplatin concentration. The process was repeated until there was certain resistance demonstrated in cells against the inhibitory behaviours of cisplatin (1000 μg/mL). A549/CR and H446/CR cells were cultivated for an extra 3 months in the culture medium that mixed RPMI‐1640 and 1000 μg/mL cisplatin. No obvious cellular morphology changes were observed in A549/CR and H446/CR cells (data not shown). Compared with parental cells, A549/CR and H446/CR cells grew more slowly (Figure 1A). A549/CR and H446/CR cells were treated with cisplatin for evaluating the cisplatin resistance behavior in them as well as in their related derivatives. As expected, cisplatin could remarkably inhibit the growth and induce apoptosis of parental cells but not resistant cells (Figure 1B,C). Furthermore, significant changes were observed in the distribution of cell cycle phases in A549/CR and H446/CR after cisplatin treatment. Cisplatin could induce G1 phase arrest strikingly in A549 and H446 cells, but not in A549/CR and H446/CR (Figure 1D). These observations demonstrate A549/CR and H446/CR cell resistance to cisplatin.

### 4.2 MiR‐130b facilitated the cisplatin resistance

**Figure 2:**
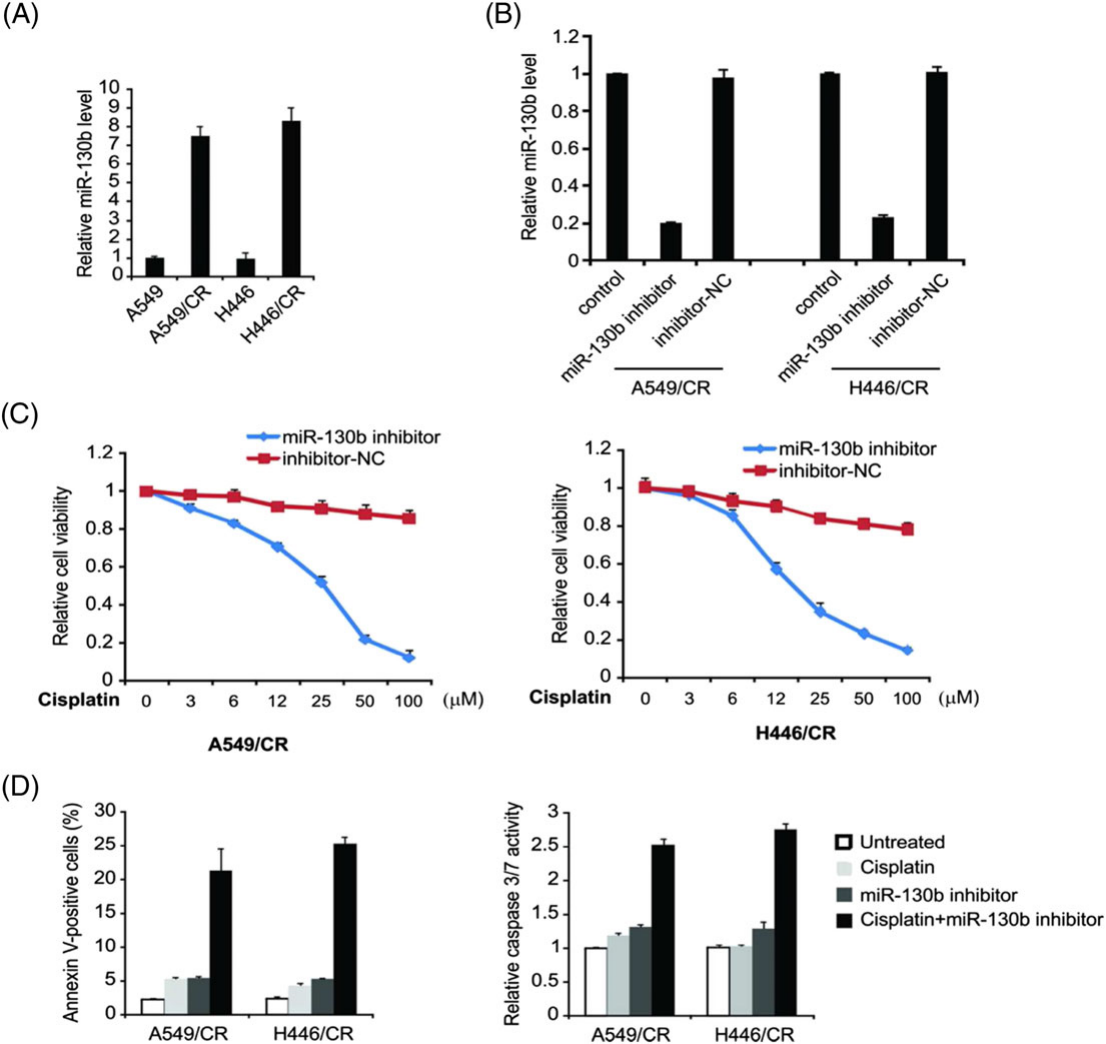
Effect of miR‐130b on resistance to cisplatin in cisplatin‐resistant lung cell lines. (A) miR‐130b level in A549 and H446 parental cells and cisplatin‐resistant cells was detected by real‐time PCR. (B) A549/CR and H446/CR cells were transfected with miR‐130b inhibitor or inhibitor‐NC for 24 hours; miR‐130b level was detected by real‐time PCR. (C) A549/CR and H446/CR cells were transfected with miR‐130b inhibitor or inhibitor‐NC for 24 hours and then treated with increasing concentrations of cisplatin for 72 hours. Cell proliferation was determined by MTS assay. (D)A549/CR and H446/CR cells were transfected with miR‐130b inhibitor or inhibitor‐NC for 24 hours and then treated with 40 μM cisplatin for 24 hours. Apoptosis was analysed by Annexin V/PI staining followed by flow cytometry (left). Caspase 3/7 activity was determined by fluorogenic analysis (right). Result in (A), (B), (C), and (D) was expressed as means ± SD of 3 independent experiments

To explore the function of miR‐130b in the cisplatin‐resistant cells A549/CR and H446/CR, we performed qRT‐PCR analysis. In comparison with parent cells, miR‐130b expression has considerable upregulation in cisplatin‐resistant cells (Figure 2A). And in order to evaluate if miR‐130b can influence the resistance against cisplatin in lung cancer cells, it was processed with miR‐130b inhibitor in A549/CR and H446/CR cell lines (Figure 2B). The viability of miR‐130b‐inhibition cells was more significantly inhibited by cisplatin compared with miR‐inhibitor‐NC cells (Figure 2C). Furthermore, miR‐130 inhibitor recovered cisplatin‐induced apoptosis in cisplatin resistance cells (Figure 2D). Thus, targeting miR‐130b reversed the cisplatin resistant behaviours in lung tumour cells.

### 4.3 MiR‐130b overexpression promotes cisplatin resistance in lung cancer cells

**Figure 3:**
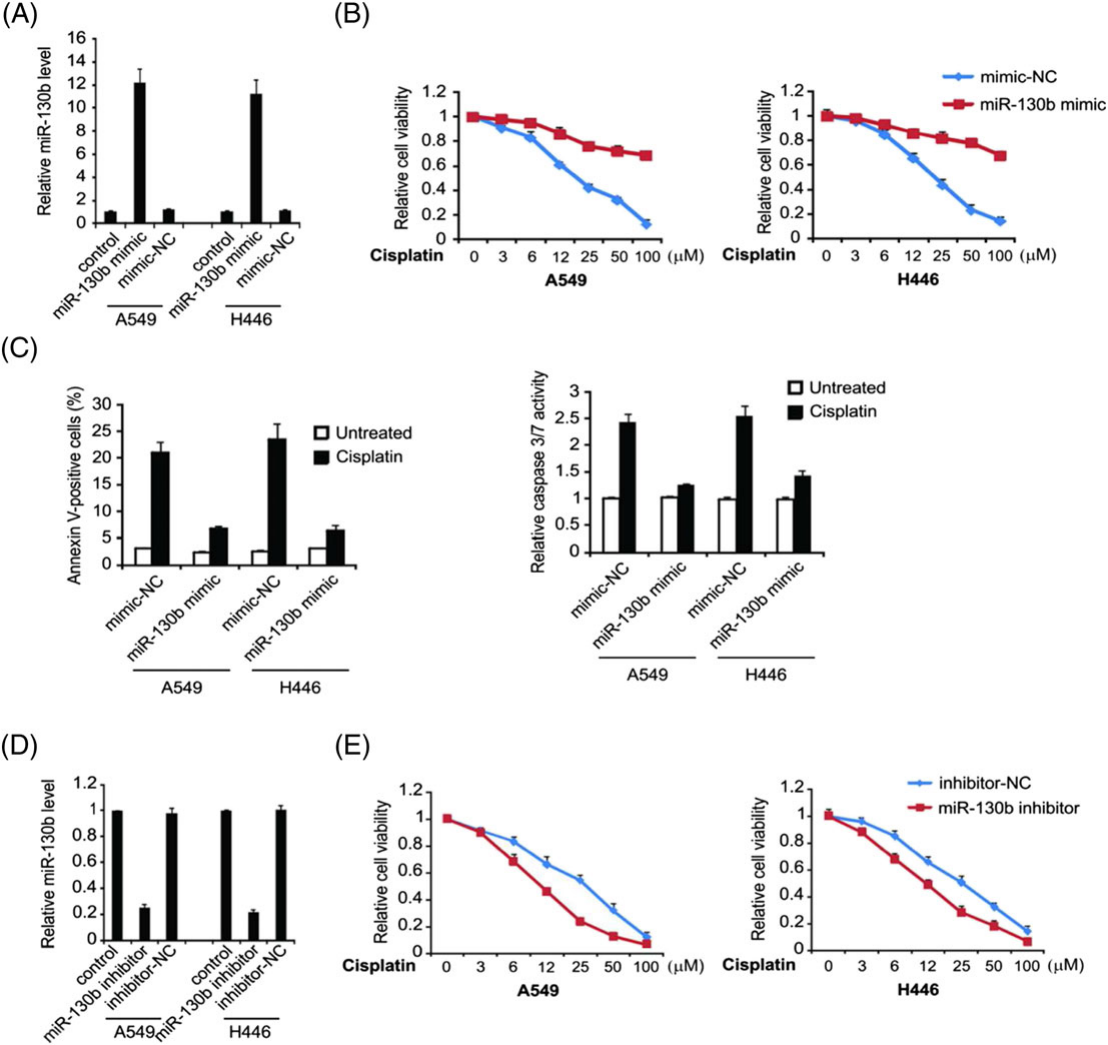
Role of miR‐130b in cisplatin resistance. (A) A549 cells were transfected with miR‐130b mimic or mimic‐NC for 24 hours; miR‐130b level was determined by real‐time PCR. (B) A549 cells were transfected with miR‐130b mimic or mimic‐NC for 24 hours and then treated with increasing concentrations of cisplatin for 72 hours. Cell proliferation was determined by MTS assay. (C) A549 cells were transfected with miR‐130b mimic or mimic‐NC for 24 hours and then treated with 40‐μM cisplatin for 24 hours. Apoptosis was analysed by Annexin V/PI staining followed by flow cytometry (left). Caspase 3/7 activity was determined by fluorogenic analysis (right). (D) A549 and H446 cells were transfected with miR‐130b inhibitor or inhibitor‐NC for 24 hours; miR‐130b level was determined by real‐time PCR. (E) A549 and H446 cells were transfected with miR‐130b inhibitor or inhibitor‐NC for 24 hours and then treated with increasing concentrations of cisplatin for 72 hours. Cell proliferation was determined by MTS assay. Results in (A), (B), (C), (D), and (E) were expressed as means ± SD of 3 independent experiments

For evaluating the regulatory impact of miR‐130b on cisplatin resistance in A549 and H446 cells, we experimented on the cisplatin sensitive cells with overexpressed miR‐130b. qRT‐PCR demonstrated that miR‐130bm greatly improved the expression level ofmiR‐130b, indicating successful transfection of miR‐130b in cells (Figure 3A). In comparison with the NC group, the A549 and H446 cells that transfected with miR‐130b mimic are more resistant to cisplatin (Figure 3B). The cisplatin‐induced apoptosis rate of miR‐130bm‐transfected A549 and H446 cells was obviously much lower than that of the NC group (Figure 3C). For continuous exploration on the influence of miR‐130b on cisplatin‐induced apoptosis in lung cancer, miR‐130 inhibitor was transfected with A549 and H446 cells. qRT‐PCR showed that the miR‐130b inhibitor downregulated miR‐130b expression of these cells (Figure 3D). Inhibition of miR‐130b significantly enhanced cells' sensitivity towards cisplatin (Figure 3E). All above results suggested that miR‐130b could facilitate lung cancer cells' resistance towards cisplatin.

### 4.4 MiR‐130b mediates PTEN protein expression via targeting 3′UTR in lung cancer

**Figure 4:**
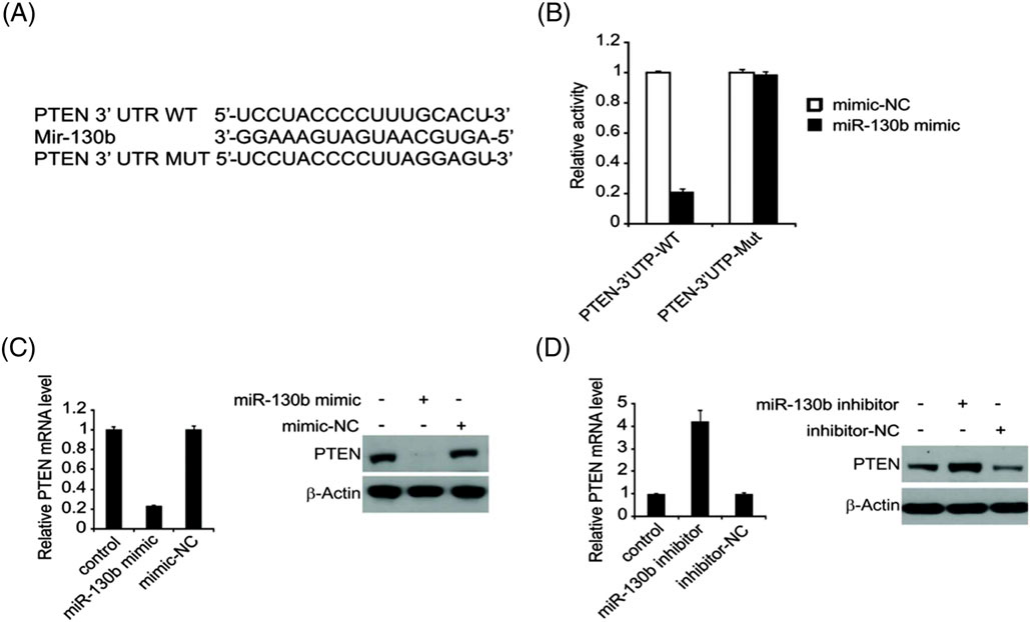
PTEN is a direct target gene of miR‐130b in lung cancer. (A) The predicted binding site of miR‐130b in the 3′‐UTR of PTEN. (B) The relative luciferase activity of A549 cells was detected after PTEN‐3′‐UTR WT or Mut were co‐transfected with miR‐130b mimic and mimic‐NC. (C) A549 cells were transfected with miR‐130b mimic or mimic‐NC for 24 hours, PTEN mRNA was detected by real‐time PCR (left), and PTEN protein level was analysed by western blotting. (D) A549 cells were transfected with miR‐130b inhibitor or inhibitor‐NC for 24 hours, mRNA was detected by real‐time PCR (left), and PTEN protein level was analysed by western blotting

PTEN gene was regarded as a target of miR‐130b (Target Scan, http://www.targetscan.org) (Figure 4A). To prove this, a luciferase reporter gene vector was built with the downstream target luciferase gene, PTEN‐3′‐UTR‐WT and PTEN‐3′‐UTR‐Mut. Afterwards, the vector underwent transfection with miR‐130b mimic and mimic‐NC each in A549 cells, where we observed a major decline in PTEN‐3′‐UTR‐WT and miR‐130b mimic group in comparison with the PTEN‐3′‐UTR‐Mut and miR‐130b mimic group (Figure 4B). All the above suggested that PTEN is miR‐130b's target gene. Next, we examined if miR‐130b mediated PTEN protein expression in A549 cells. Data showed that transfected miR‐130b mimic effectively suppressed the protein expression of PTEN mRNA in A549 cells (Figure 4C). PTEN protein and mRNA were upregulated in the miR‐130b inhibitor‐transfected A549 cells (Figure 4D). The experiment outcomes demonstrated an affirmative inverse relationship between the levels of miR‐130b and PTEN.

### 4.5 Overexpression of miR‐130b activates Wnt/β‐catenin pathway through PTEN inhibition

**Figure 5:**
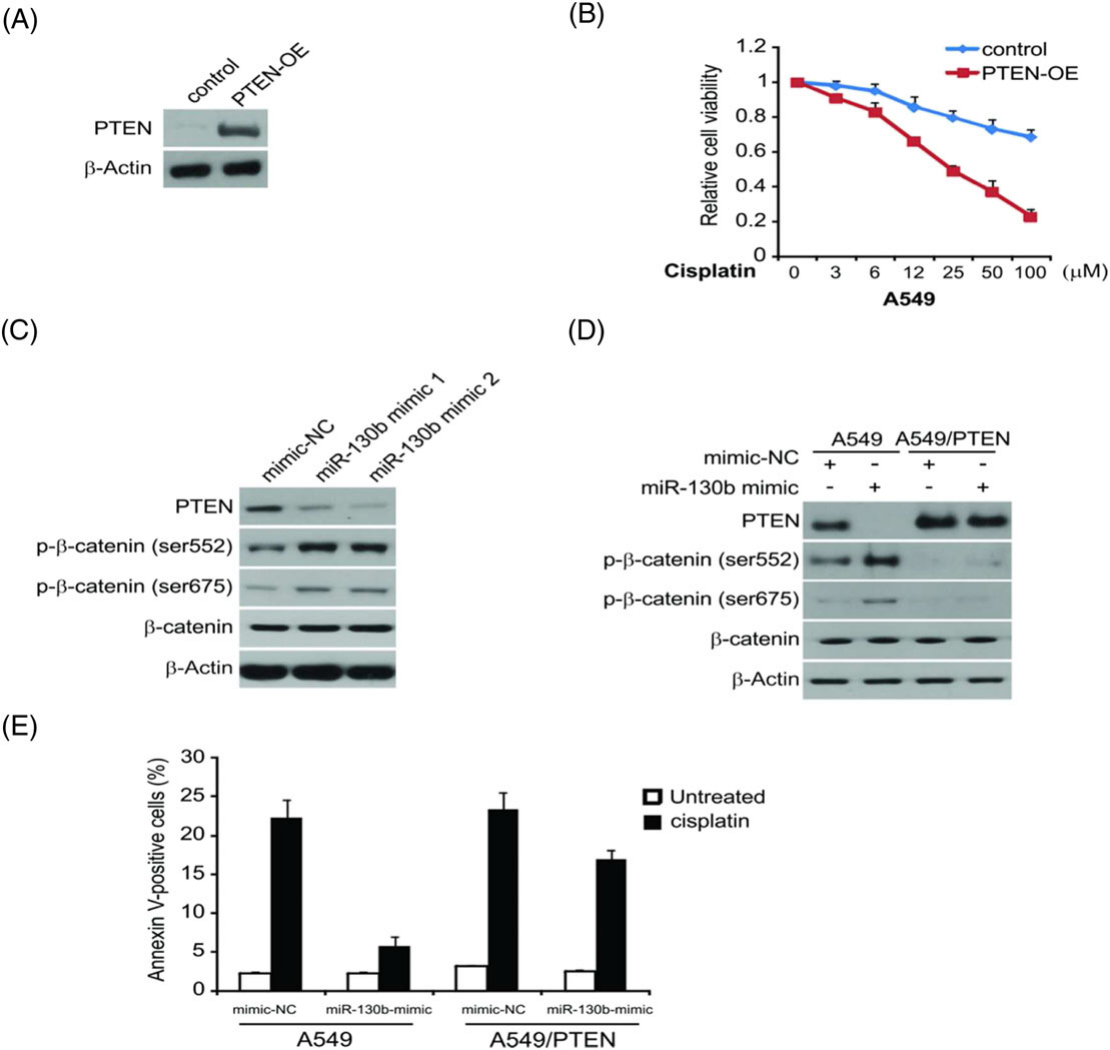
miR‐130b promotes cisplatin resistance via Wnt/β‐catenin signalling pathway. (A) A549 cells transfected with miR‐130b mimic with or without PTEN plasmid co‐transfection, PTEN expression were analysed by western blotting. (B) A549 cells transfected with miR‐130b mimic with or without PTEN plasmid co‐transfection were treated with increasing concentrations of cisplatin for 72 hours. Cell proliferation was determined by MTS assay. (C) A549 cells were transfected with miR‐130b mimic or mimic‐NC for 24 hours, indicated protein level was analysed by western blotting. (D) A549 and A549/PTEN cells were transfected with miR‐130b mimic or mimic‐NC for 24 hours, indicated protein level was analysed by western blotting. (E) A549 and A549/PTEN cells were transfected with miR‐130b mimic or mimic‐NC for 24 hours and then treated with 40 μM of cisplatin for 24 hours

In order to determine the function of PTEN on cisplatin resistance mediated by miR‐130b, PTEN plasmid or control vector was processed into the miR‐130b mimic‐transfected cisplatin‐resistant A549 cells to measure the cell viability in different cisplatin concentration conditions. Western blotting analysis indicated that PTEN overexpression greatly upregulated PTEN's protein level (Figure 5A) and also considerably decreased the survival rate of the A549 cisplatin resistance cells than in the NC group (Figure 5B). This indicated that miR‐130b may regulate cisplatin resistance in the A549 cisplatin resistance cells via reducing PTEN protein level.

In addition, Western blotting analysis demonstrated that the miR‐130b overexpression reduced the PTEN expression and decreased the phosphorylation of downstream kinase β‐catenin (Figure 5C). PTEN has the ability of suppressing various types of tumours and is a negative regulating factor of Wnt/β‐catenin.^18^ Hence, we assumed that miR‐130b's overexpression could activate Wnt/β‐catenin pathway via cutting down PTEN's protein level, which led to A549 cells' resistance to cisplatin. For further exploration of the impact of Wnt/β‐catenin pathway on miR‐130b‐induced cell survival and cisplatin resistance, we treated the miR‐130b mimic transfected A549 or A549/PTEN cells with cisplatin in different dosages. Findings demonstrated that PTEN abrogated partly the phosphorylation of β‐catenin triggered by miR‐130b transfection (Figure 5D). Meanwhile, miR‐130b‐induced cisplatin resistance was greatly suppressed (Figure 5E). All the above indicated that miR‐130b overexpression was able to activate the Wnt/β‐catenin pathway through downregulating PTEN in lung cancer cells.

### 4.6 Targeting miR‐130b overcome cisplatin resistance in vivo

**Figure 6:**
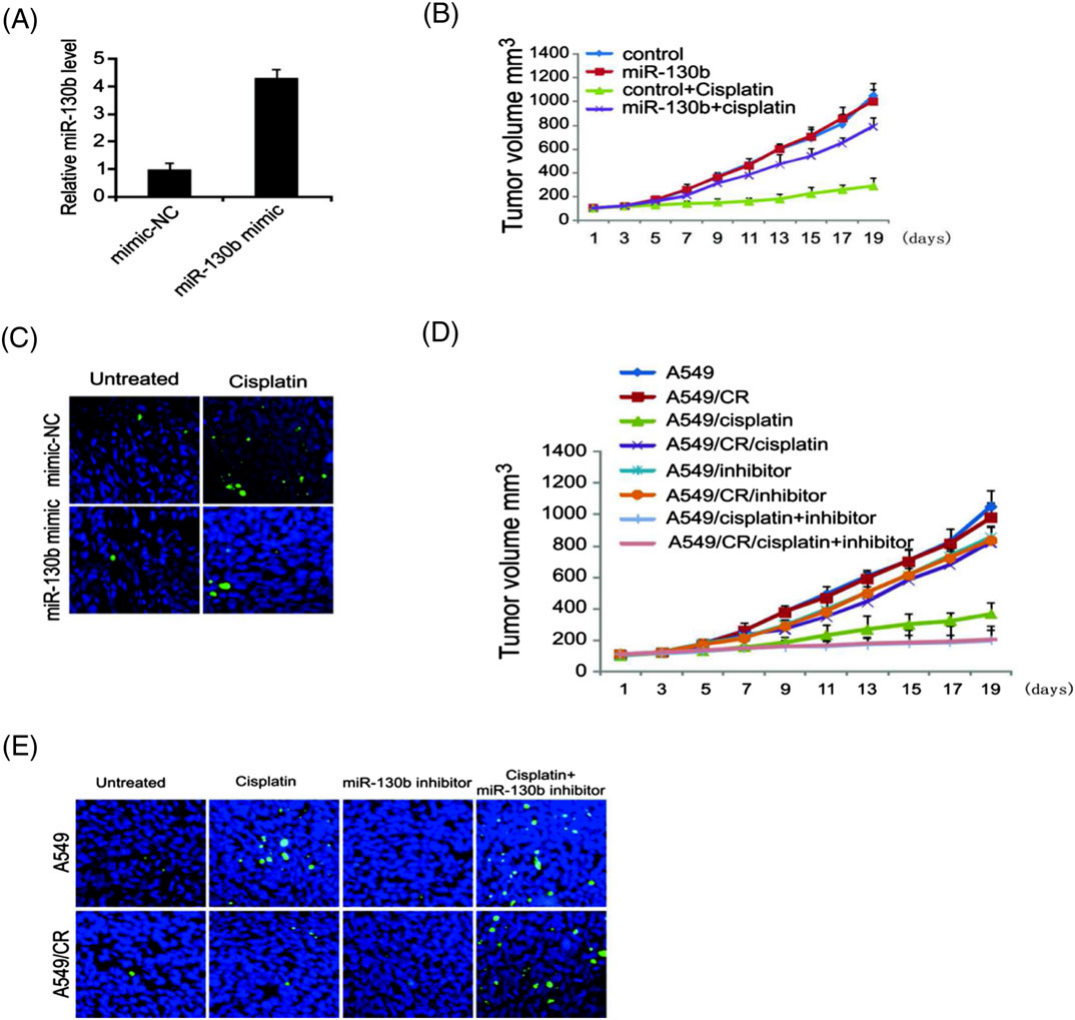
miR‐130b mediates cisplatin resistance in vivo. (A) miR‐130b expression in tumour was detected by real‐time PCR. (B) Mice were treated with cisplatin or buffer. Tumour volume was calculated after treatment (*n* = 6 in each group). (C) Paraffin‐embedded sections of tumour tissues from mice were analysed by TUNEL staining. (D) Mice were treated with cisplatin, miR‐130b, or the combination. Tumour volume was calculated after treatment (*n* = 6 in each group). (E) Paraffin‐embedded sections of tumour tissues from mice were analysed by TUNEL staining. Results in (A), (B), and (D) were expressed as means ± SD of 3 independent experiments

To evaluate the potential function of miR‐130b on cisplatin therapy internally, A549 cells with overexpressed miR‐130b were implanted in mice. The miR‐130b levels in the miR‐130b‐stable A549 cells compared with that in the routine A549 were demonstrated in Figure 6A. We discovered that the average size of tumours in the miR‐130b group was considerably larger than the EV group even though they were administrated with the same dosage of cisplatin (Figure 6B). This showed the overexpression of miR‐130b resistance in the anti‐tumour effect of cisplatin within the body. We also examined tumour cells apoptosis in xenografts, which demonstrated that cisplatin‐triggered cell apoptosis was reduced while miR‐130b was overexpressed (Figure 6C). The results showed that cisplatin coupled with miR‐130 inhibitor together could suppress the cisplatin‐resistant cell growth outside the body. To determine if this is valid as well inside the body, A549 and A549/CR tumours were administrated with cisplatin, miR‐130b inhibitor, or their combination. The combination of cisplatin with miR‐130b inhibitor more successfully suppressed the progression of tumours than cisplatin alone (Figure 6D), which meant miR‐130b could be a prognosis indicator for the combination of cisplatin and miR‐130b inhibitor in lung cancer. Tumour cell death was also aligned with the same trend (Figure 6E). The results indicated that targeting miR‐130b may assist to overcoming cisplatin resistance.

## 5 DISCUSSION

The general cause of drug resistance in tumour cells is due to accumulation of changes in genes altered the genetic pathways, thus stimulating cancer cell evolution of phenotypes.^19^, ^20^ There have been limited effective solutions to solve the resistance problem in cancer treatment over the years.^19^, ^21^ Previous researches have demonstrated that PTEN is related with the drug resistance in various cancers.^22^ Our study gave evidence to the mechanistic connection of miR‐130b upregulation in cisplatin resistance in lung cancer. Our findings suggest that miR‐130b targets PTEN to mediate chemoresistance, proliferation, and apoptosis via Wnt/β‐catenin pathway in lung cancer cells.

Lately, miRNAs have been shown as a crucial regulator for the cellular responses of tumour cells against treatment.^23^ Patients' response to chemotherapy has been proved to connect tightly with the functioning of miRNAs.^24^, ^25^ Although its underlying mechanism still remains unsure, experiments and observations have so far revealed several possibilities of roles that miRNAs could play, such as changing the targets of certain drug, affecting therapy‐induced apoptosis, mediating proteins involved in multiple drug resistance, and stimulating angiogenesis activities, etc. In our research, we discovered that miR‐130b upregulated A549/CR cells in comparison with the A549 cells experiment group. The findings revealed that overexpression of miR‐130b encouraged resistance to cisplatin in lung cancer cells, and the inhibition of miR‐130b overexpression could reverse cisplatin resistance. PTEN was viewed to be 1 target of miR‐130b, and its signalling pathway has been recorded to regulate a variety of cellular activities in tumour settings including programmed cell death, cell proliferation, invasion, etc. In this sense, the hypothesis of miR‐130b targeting PTEN is in accordance with its acknowledged biological impact. Actually, certain miRNAs can assist resistance via targeting PTEN in different tumours.

PTEN loss is a commonly seen genetic variation in cancers like, gastric cancer, breast cancer, and spongioblastoma.^26^, ^27^ It is closely connected with cytotoxic drug resistance. Our study suggested that miR‐130b mediated the gene expression of PTEN in lung cancer cells with a negatively correlation. Consequently, the aggressiveness of these cells was intensified after the miR‐130b expression transfection process. Increasing evidences demonstrated that PTEN malfunction had prognostic implications in some malignant tumours like lung cancer. Our research findings showed that targeting PTEN at posttranscriptional stage via miRNAs, eg, miR‐130b could regulate its downregulation process. All the findings indicated that elevated miR‐130b expression could be related with diminishing survival rate of lung cancer patients and the miR‐130b inhibitor bears possible capability of countering drug resistance. Reducing miR‐130b's level may effectively increase the sensitivity of tumour cells to cisplatin. We expect treatment strategy to be developed based on miR‐130b levels. Combination cisplatin with the inhibition of miR‐130b could enhance the sensitivity and effectiveness of drugs in lung cancer. In addition, we revealed that miR‐130b reacts to cisplatin resistance in lung cancer cells via altering its target PTEN's level and downstream Wnt/β‐catenin signalling pathway. Wnt/β‐catenin signal pathway is among the most crucial signalling pathways during embryonic development and remains a popular problem for tumour research.^28^ Recently, accumulating evidence reported the relevance of Wnt/β‐catenin signal pathway in the chemoresistance of various cancers.^29^ Hereby, we revealed that Wnt/β‐catenin has a key influence on miR‐130b mediated cisplatin resistance.

## 6 CONCLUSIONS

Our results uncovered the unique function of miR‐130b in cisplatin‐resistance, which was done mainly through activating Wnt/β‐catenin signalling pathway of lung tumour. Although the resistance to drug is a main problem for the lung cancer therapy involved cisplatin, the method for inhibition of the miR‐130b could be a novel way to improve the sensitivity of lung cancer to cisplatin.

## CONFLICT OF INTERESTS

None declared.

## FUNDING

None declared.
